# How to detect and track chronic neurologic sequelae of COVID-19? Use of auditory brainstem responses and neuroimaging for long-term patient follow-up

**DOI:** 10.1016/j.bbih.2020.100081

**Published:** 2020-05-15

**Authors:** Michael Ogier, Guillaume Andéol, Emmanuel Sagui, Gregory Dal Bo

**Affiliations:** a French Armed Forces Biomedical Research Institute, 1 place Valérie André, 91220, Brétigny sur Orge, France; b European Hospital of Marseille, 6 rue Désirée Clary, 13003, Marseille, France

**Keywords:** COVID-19, SARS-CoV-2, Neurologic sequelae, Neuroinflammatory mechanisms, Cytokine storm, Microglia priming, Brainstem dysfunction, Multiple sclerosis, Auditory brainstem responses, Neuroimaging

## Abstract

This review intends to provide an overview of the current knowledge on neurologic sequelae of COVID-19 and their possible etiology, and, based on available data, proposes possible improvements in current medical care procedures. We conducted a thorough review of the scientific literature on neurologic manifestations of COVID-19, the neuroinvasive propensity of known coronaviruses (CoV) and their possible effects on brain structural and functional integrity. It appears that around one third of COVID-19 patients admitted to intensive care units (ICU) for respiratory difficulties exhibit neurologic symptoms. This may be due to progressive brain damage and dysfunction triggered by severe hypoxia and hypoxemia, heightened inflammation and SARS-CoV-2 dissemination into brain parenchyma, as suggested by current reports and analyses of previous CoV outbreaks. Viral invasion of the brain may particularly target and alter brainstem and thalamic functions and, consequently, result in sensorimotor dysfunctions and psychiatric disorders. Moreover, data collected from other structurally homologous CoV suggest that SARS-CoV-2 infection may lead to brain cell degeneration and demyelination similar to multiple sclerosis (MS). Hence, current evidence warrants further evaluation and long-term follow-up of possible neurologic sequelae in COVID-19 patients. It may be particularly relevant to evaluate brainstem integrity in recovered patients, as it is suspected that this cerebral area may particularly be dysfunctional following SARS-CoV-2 infection. Because CoV infection can potentially lead to chronic neuroinflammation and progressive demyelination, neuroimaging features and signs of MS may also be evaluated in the long term in recovered COVID-19 patients.

## Highlights

• Acute neurologic symptoms are observed in one third of COVID-19 patients.
• Neurotropism of SARS-CoV-2 is highly suspected.
• Brainstem damage may be a crucial driver for COVID-19-associated respiratory failure.
• COVID-19 is likely to trigger demyelination similarly to multiple sclerosis.
• Monitoring patients using Auditory Brainstem Responses and neuroimaging is warranted.

## 1 Introduction

In December 2019, a new human coronavirus (HCoV) infection has emerged in Wuhan, China. Unlike recent severe acute respiratory syndrome (SARS)-CoV-1 and Middle East respiratory syndrome (MERS)-CoV outbreaks, the recent SARS-CoV-2/COVID-19 pandemic has spread quickly and has been reported in more than 200 countries worldwide. The ongoing epidemic has already caused over 240,000 deaths worldwide, mostly due to respiratory failure, the death rate being around 3–4% of infected individuals (reported on May 2, 2020 by the European CDC). HCoV are known to affect several organs including the brain (Gu et al., 2005) and may be responsible for several neurologic complications (Lau et al., 2004; Arabi et al., 2015). Acute neurologic symptoms have already been reported in COVID-19 patients (Mao et al., 2020), and their mechanisms have recently been discussed (Wu et al., 2020). In addition, a case of meningoencephalitis associated with presence of SARS-CoV-2 RNA in cerebrospinal fluid (CSF) samples was recently reported (Moriguchi et al., 2020). This evidence raises the possibility that SARS-CoV-2 might invade the brain and cause acute and progressive neurologic deficits, as already suspected by others (Troyer et al., 2020). Therefore, it may be important to anticipate and track potential long-term sequelae of COVID-19.

The neuroinvasive potential of SARS-CoV-2 and its consequences on brain function are currently a subject of great debate. Unfortunately, there is a great paucity of information available on the effects of SARS-CoV-2 on brain integrity at the present moment. Nevertheless, current clinical and preclinical data warrant improvements in patient care practices. In particular, non-invasive evaluation of neurologic functions and of brain inflammatory status may be implemented in routine clinical protocols. In this review article, we expose the current knowledge on how HCoV infection can lead to cerebral dysfunction and how it may relate to disease. We also discuss few complementary clinical examinations that could potentially alleviate COVID-19 patient monitoring and care.

## 2 Neurologic manifestations of COVID-19

### 2.1 Clinical presentation

In one of the first retrospective studies published, 36.4% (78/214) of Chinese COVID-19 patients exhibited neurologic symptoms, i.e. headache, dizziness and loss of consciousness (Mao et al., 2020). There is accumulating evidence of neurologic symptoms in individual COVID-19 patients: meningoencephalitis (Moriguchi et al., 2020), acute hemorrhagic necrotizing encephalopathy (Poyiadji et al., 2020), acute myelitis and Guillain-Barre syndrome (Zhao et al., 2020). Moreover, a very recent study showed that 84% of patients (49/58) admitted to intensive care units (ICU) in Strasbourg (France) due to acute respiratory distress syndrome (ARDS) exhibited neurologic symptoms, including agitation, confusion and signs of corticospinal tract dysfunction, during medical care (Helms et al., 2020). At discharge, 33% of patients (15/45) had suffered from dysexecutive symptoms (e.g. inattention, disorientation, or poorly organized movements in response to command). Of 13 patients who underwent MRI neuroimaging, 62% had signs of meningitis, 100% had perfusion abnormalities, and 23% had a cerebral ischemic stroke. Unlike other reports (Moriguchi et al., 2020), CSF samples collected from 7 patients were negative for SARS-CoV-2.

### 2.2 Etiology

The etiology of these neurologic manifestations is still unclear. Some of the symptoms reported, especially headache, dizziness and loss of consciousness, are not specific, as they could possibly be due to the severe hypoxia associated with respiratory failure. Other symptoms could result from the dysimmune state caused by the infection, in particular from the abnormally high inflammation that is referred to as “cytokine storm”. For instance, cytokine storm may be involved in acute neurovascular pathologies observed in COVID-19 patients without pre-existing vascular risk factors (Talan, 2020). Moreover, the occurrence of cerebral hemorrhage in COVID-19 patients may be facilitated by SARS-CoV-2 interaction with its cellular receptor, the angiotensin-converting enzyme 2 (ACE2) (Wang et al., 2020). Indeed, dysfunction of ACE2 due to its interaction with the virus may lead to hypertension. This hypothesis is corroborated by a recent study that demonstrated that anticoagulant treatment can improve survival of COVID-19 patients (Tang et al., 2020). Olfactory dysfunction, i.e. anosmia, has been reported in up to 85.6% of mild-to-moderate COVID-19 patients (Lechien et al., 2020). Anosmia may be a sign of olfactory bulb viral infection, which could represent the first step through which SARS-CoV-2 invades the brain, through olfactory neurons, as this dissemination route has already been described in preclinical studies of SARS-CoV-1 (Netland et al., 2008).

## 3 Neuroinvasion of SARS-CoV-2 and neurologic symptoms: potential routes of dissemination

### 3.1 Neuronal route

Even if there is still sparse evidence of neuroinvasion by SARS-CoV-2, viral particles have been detected in the CSF of one infected patient with meningoencephalitis (Moriguchi et al., 2020) and in the frontal lobe of one patient with Parkinson’s disease who exhibited fever and confusion at admission (Paniz-Mondolfi et al., 2020). Animal studies have shown that two HCoV, HCoV-OC43 and SARS-CoV-1, can penetrate the CNS via the olfactory nerves (Desforges et al., 2019). A path from the olfactory bulb to the brainstem through the piriform cortex has been demonstrated in transgenic mice expressing human ACE2 and intranasally inoculated with SARS-CoV-1 (Netland et al., 2008). Similarly, HCoV-OC43 can infect the olfactory bulb of mice after nasal inoculation and can spread into the brain via transneuronal transport up to the spinal cord (St-Jean et al., 2004; Desforges et al., 2019). HCoV-OC43 disseminates to the whole brain in less than 7 days and can be detected in neurons as well as glial cells (St-Jean et al., 2004). Other neuronal routes have been described: for instance, the influenza A virus can reach brainstem respiratory centers from the lungs via the vagus nerve (Matsuda et al., 2004).

### 3.2 Hematogenous route

As previously described for SARS-CoV-1 and HCoV-OC43, SARS-CoV-2 may also reach the brain via an hematogenous route (Desforges et al., 2019). It is suspected that SARS-CoV-1 can take advantage of the permeabilization of the blood-brain barrier (BBB) caused by excessive amounts of cytokines and chemokines released after viral infection of the airways (McCray et al., 2007). MERS-CoV could also use the leukocytes as “Trojan horses” to penetrate the CNS. Indeed, activated leukocytes express the enzyme dipeptidyl-peptidase 4 (DPP4), which is a functional receptor of MERS-CoV (Zhao et al., 2015). The hypothesis that MERS-CoV may use the hematogenous route to reach the brain is supported by the fact that some neurologic symptoms (seizure, encephalitis, stroke) are reported to emerge 2–3 weeks after ARDS (Kim et al., 2017). Nevertheless, in the first days following infection, the hypothesis of hematogenous route is debated. SARS-CoV-1 and MERS-CoV are not detected within the brain in non-neuronal cells, suggesting a more direct pathway to reach the brain (Li et al., 2020). In addition, in a mouse model of hepatitis virus infection, viral antigens were still not detected in blood cells 3 days after intranasal inoculation while they were already present in olfactory bulbs (St-Jean et al., 2004).

### 3.3 Target cells

As for SARS-CoV-1 virus, the cellular receptor for SARS-CoV-2 is the ACE2 (Hoffmann et al., 2020). The enzyme is highly expressed in the heart, kidney and vasculature (Harmer et al., 2002). It has also been detected in several peripheral tissues, including the nasopharynx, lung, stomach, small intestine, colon, skin, lymph node, thymus, bone marrow, spleen and liver (Hamming et al., 2004). In addition, ACE2-mRNA and protein have been detected in several regions of the brain (Harmer et al., 2002; Hamming et al., 2004; Xia and Lazartigues, 2008). The typology of ACE2-expressing brain cells is still debated (Xia and Lazartigues, 2008). However, animal studies suggest that the enzyme is predominantly expressed by neurons (Doobay et al., 2007). Hence, neurons might be directly targeted and infected by SARS-CoV-1 and SARS-CoV-2.

## 4 Central nervous system pathophysiology

### 4.1 Neuroinflammatory mechanisms

#### 4.1.1 Cytokine storm

Several mechanisms may contribute to CNS damage after HCoV infection: through direct viral replication in infected cells (neuropathology) or through an indirect process involving neuro-immunopathology. Different studies have shown that systemic inflammation along with massive release of cytokines (i.e. “cytokine storm”) contribute to brain damages induced by SARS-CoV-1 (Xu et al., 2005; Li et al., 2016b). Similar exacerbated inflammatory status was also reported in SARS-CoV-2 infected patients, and was characterized by elevated blood concentrations of interleukin (IL-)6, 8 and 10 and of tumor necrosis factor alpha (TNF-α) (Chen et al., 2020b, 2020a). Inflammation was more prominent in severe than in moderate COVID-19 cases, and in deceased victims more than in recovered patients, suggesting that inflammatory processes play a critical role in disease progression and mortality after SARS-CoV-2 infection (Chen et al., 2020b, 2020a). In addition, peripheral lymphopenia was detected in the majority of COVID-19 patients regardless of clinical severity (Chen et al., 2020a). All patients with impaired inflammatory status exhibited respiratory failure and few of them were diagnosed with hypoxic encephalopathy, suggesting that SARS-CoV-2 infection can affect the CNS (Chen et al., 2020a).

Few reports suggest that the cytokine storm may reach the CNS. Indeed, elevated cytokine concentrations have been detected in the CSF of HCoV infected patients (Li et al., 2016b). Granulocyte-macrophage colony-stimulating factor, pro-inflammatory cytokines (IL-6 and IL-8) and monocyte chemoattractant protein levels were significantly elevated in the CSF of HCoV patients, whereas blood levels of these inflammatory molecules were close to control ranges (Li et al., 2016b). Moreover, histologic evaluations of brains from patients that died from SARS-CoV-1 infection have shown foci of necrotic cell death, edema, glial scar and infiltrated immune cells (macrophages and T lymphocytes) (Gu et al., 2005; Xu et al., 2005; Guo et al., 2008). Hence, HCoV infection is likely to result in increased brain inflammation. However, the underlying mechanisms have yet to be elucidated.

To further investigate the cerebral effects of SARS-CoV-1 infection, a transgenic mouse line expressing the Human ACE2 was engineered (McCray et al., 2007; Netland et al., 2008). Expression of hACE2 makes these mice prone to brain invasion by SARS-CoV-1. Sixty hours after intranasal inoculation, SARS-CoV-1 viral particles are detected in olfactory bulbs (the most infected region) and in deeper brain parts, including the piriform and infralimbic cortices, basal ganglia, dorsal raphe, thalamic nuclei and brainstem (Netland et al., 2008). Unlike reports in human brain samples, histologic evaluations in these mice showed restricted brain inflammation without change in astrocyte density, but with an increase in the density of microglial cells (Netland et al., 2008). In addition, significant elevation of IL-6, CCL2, IFN-γ and CCL12 expression was measured in mice brain 4 days after SARS-CoV-1 infection (McCray et al., 2007). Hence, these experimental studies corroborate the hypothesis that HCoV infection may lead to brain inflammation. Similar results were obtained in murine models infected with mouse hepatitis virus (MHV). Following intranasal inoculation with the JHM (John Howard Muller) strain of MHV, microglial cells are quickly activated, phagocytize infected cells and release several cytokines including IFN-α/β, CCL2, TNFα and IL-6 (Wheeler et al., 2018). This suggests that brain immune response to CoV infection is similar regardless of the viral strain and suggest a predominant role of microglia in the initial stage of defense reaction to CoV infection. This hypothesis is corroborated by the fact that depletion of microglia exacerbates viral replication and is associated with faster viral spread in the brain of infected mice (Wheeler et al., 2018). Moreover, it seems that microglia recruitment is required for proper activation of T lymphocytes after infection (Wheeler et al., 2018).

Absent from the brain in homeostatic conditions, CD4^+^ and CD8^+^ T lymphocytes can invade the CNS when the BBB is permeabilized by metalloproteinases secreted by neutrophils and macrophages (Skinner et al., 2019). Six days after JHMV inoculation, both types of T lymphocytes are found in mice brains, with the highest cell infiltration occurring between 7 and 10 days post-inoculation (Phares et al., 2013). Based on their cytolytic activity, CD8^+^ T cells are likely to play a fundamental role in control and clearance of the virus, while CD4^+^ T cells may locally enhance the immune response by releasing IFN-γ and stimulating the expression of major histocompatibility complex (MHC) proteins in microglial cells (Wheeler et al., 2018). These mechanisms are most probably triggered in order to increase viral clearance, since it requires both MHC 1 and 2 expression by immunocompetent cells (Skinner et al., 2019). Two cytokines seem to be fundamental for initiation of the inflammatory reaction following JHMV infection. A sentinel role for CXCL10 is suggested, as its release attracts T lymphocytes (Skinner et al., 2019), while IL-21 may optimize both B and T lymphocyte responses in the CNS (Phares et al., 2013). In addition, CD4^+^ regulatory T cells (Treg) are recruited when inflammatory response reaches a peak, and modulate the extend of the immune defense response (de Aquino et al., 2013). Treg are essential to avoid subsequent damage induced by prolonged neuroinflammation, and their action also limit viral replication, although it does not influence the persistence of the virus in the CNS (de Aquino et al., 2013). These data suggest that if cerebral inflammation is contained, CoV-induced neuropathologies may be avoided or, at least, alleviated.

#### 4.1.2 Microglia priming as a possible aggravating factor of post-infectious neuroinflammation

Microglial cells are the first endogenous immune responders after brain infection or traumatic injury, as their primary function is to maintain cerebral homeostasis and avoid tissue degradation (Hanisch and Kettenmann, 2007; Ransohoff and Perry, 2009). When they are activated, microglial cells produce and release several inflammatory chemo-mediators (pro-inflammatory cytokines and chemokines) and several reactive oxygen species (ROS) (Ransohoff and Perry, 2009; Chatterjee et al., 2013), and they coordinate inflammatory responses between the CNS and the peripheral immune system. After activation, microglia can become phagocytic, remove injured cells and clear cellular debris, and are able to present antigens to T lymphocytes (Wheeler et al., 2018). Microglial activation is typically downregulated when cerebral homeostasis is restored.

Under certain circumstances, endogenous brain immune processes may be chronically activated. It was particularly pointed out that chronic stress, traumatic brain injury, neurodegenerative diseases and aging are all associated with profound and chronic changes in the nature and protective role of microglia (Norden et al., 2015; Fonken et al., 2018). This phenomenon is called microglial “priming”. Primed microglia are more reactive to immune challenges, releasing abnormally high amounts of pro-inflammatory cytokines for extended durations. Exacerbated microglia activation can lead to extended lesions after physical brain trauma, and interfere with brain function (Norden et al., 2015). Microglia priming has been linked with a large number of severe neuropsychiatric disorders, including addiction, major depressive disorder, bipolar disorder and schizophrenia (Tay et al., 2017). Exaggerated reactivity of primed microglia may, therefore, be particularly detrimental in the context of SARS-CoV-2 infection, as COVID-19 is associated with intense systemic immune reaction (the so-called “cytokine storm”). On the other hand, excessive and long-lasting immune reaction pertaining to SARS-CoV-2 infection may prime microglia and, therefore, be responsible for development of chronic neuropsychiatric symptoms.

### 4.2 Respiratory symptoms caused by SARS-CoV-2 infection: possible association with brainstem dysfunction

So far, most studies on SARS-CoV-2 have focused on its effects on the respiratory tract. Most patients initially present with dyspnea and develop pneumonia that can lead to ARDS, and ultimately, to death (Chen et al., 2020b; Huang et al., 2020; Zhu et al., 2020). Nevertheless, etiology of these respiratory deficits remains to be fully elucidated. The type of pneumonia associated with infection with SARS-CoV-2 has rarely been described in great details, although pneumonia can have very diverse causes. Aspiration pneumonia, for example, may result from dysfunction of the brain rather than the respiratory tract (Balofsky et al., 2017). A very recent study reported the case of a SARS-CoV-2 infected patient who died from acute bacterial bronchopneumonia most probably caused by aspiration (Barton et al., 2020). Unfortunately, his CNS was only grossly examined and was reported as being healthy. The vast majority of radiologic examination reports published so far have described the same overall pattern of pulmonary lesions in COVID-19 patients, i.e. ground-glass opacities and consolidations, which seem to be temporally linked with the evolution of respiratory symptoms (Shi et al., 2020; Zhao et al., 2020; Zhou et al., 2020). Interestingly, these types of lesions may not necessarily be due to deterioration of the lungs following direct viral infection, as they are also frequently reported in dysphagic patients with chronic aspiration that are particularly susceptible to aspiration pneumonia (Scheeren et al., 2016). A retrospective study on 113 deceased COVID-19 patients revealed that 44% of them initially presented with dyspnea and 72% developed ARDS (Chen et al., 2020b). ARDS was mainly associated with type 1 respiratory failure, which most probably originated from lung deterioration and was also associated, although to a much lesser extent, to signs of type 2 respiratory failure, which can result from extra-pulmonary deficits, especially from brainstem dysfunction. Hence, SARS-CoV-2-related respiratory failure may be multifactorial and result from dysfunction of multiple organs.

A large body of evidence suggests that respiratory failure linked with SARS-CoV-2 infection might be, at least in part, due to brain dysfunction. Indeed, number of animal studies have pointed out that brainstem neurons are targeted by CoV and may subsequently die. This may result in respiratory failure, as brainstem neurons are in charge of the regulation of most vital physiological processes, especially of respiratory and cardiac functions (Nicholls and Paton, 2009).

Studies performed in the early 80’s in piglets have demonstrated that infection with CoV PHEV, which shares high structural homology with human HCoV-OC43, leads to neurologic symptoms and vomiting diseases associated with encephalomyelitis (Andries and Pensaert, 1980). These studies have demonstrated that after oronasal inoculation, CoV PHEV could be detected in the brain of piglets, especially in the brainstem region (Andries and Pensaert, 1980). The brainstem is rapidly infected by CoV PHEV, which is initially detected in medullary sensory trigeminal and vagal neurons. These neurons are particularly involved in regulation of the digestive tract function, deglutition, as well as the cardiac and respiratory functions (Diamant, 1997; Browning and Travagli, 2014; Benarroch, 2018). It is likely that the vomiting diseases pertaining to CoV PHEV infection be the consequence of lesions located within these medullary brainstem nuclei and the resulting dysfunction of the digestive tract.

Other sets of data, mostly collected from rodent studies, support the hypothesis that respiratory deficits triggered by infection with CoV may, to some extent, have a cerebral origin. K18-hACE2 mice, a transgenic mouse line expressing the human ACE2 gene under the cytokeratin 18 promoter, were used to experimentally study CoV infection (McCray et al., 2007; Netland et al., 2008). After intranasal inoculation with SARS-CoV-1, K18-hACE2 mice develop many symptoms found in human patients, including loss of weight, lethargy and labored breathing. Infected mice are severely ill as they usually die within 5–7 days following inoculation. Experimental studies demonstrated initial lung infection followed by secondary viral spread to the brain. In particular, presence of viral particles has clearly been detected within the thalamus and brainstem regions of transgenic mice. Among other brain structures, medullary nuclei of the dorsal vagal complex, i.e. the nucleus of the solitary tract, area postrema, and the motor nucleus of the vagus nerve, along with nucleus ambiguous, were listed (Netland et al., 2008). All these brainstem nuclei are critical for neural control of the cardiac and respiratory functions (Benarroch, 2018). It is noteworthy that these nuclei seemed to be infected independently of the inoculation route, and may therefore be preferentially targeted by SARS-CoV-1. Indeed, when injected intracranially and at very low titers, SARS-CoV-1-infected cells could still be identified in the dorsal vagal complex. Moreover, the presence of lung deterioration (neutrophilic infiltration and aspiration pneumonia) with very low endogenous viral titer and absence of viral antigens after intracranial injection of SARS-CoV-1 corroborated the cerebral origin of respiratory dysfunction after viral exposure (Netland et al., 2008).

### 4.3 Brain dysfunction, neurologic symptoms, and death

Animal studies focusing on MERS-CoV infection also argue in favor of a cerebral origin for CoV-associated pathologies. Transgenic murine lines expressing the human DPP4 have been studied (Agrawal et al., 2015; Tao et al., 2015; Li et al., 2016a). After intranasal MERS-CoV inoculation, hDPP4 mice develop diverse pathologies and rapidly die, similarly to SARS-CoV-1 inoculated K18-hACE2 mice (McCray et al., 2007; Netland et al., 2008; Agrawal et al., 2015; Tao et al., 2015; Li et al., 2016a). These studies confirmed the viral neurotropism of MERS-CoV. MERS-CoV infection is associated with sharp increase in brain inflammatory markers (Tao et al., 2015; Li et al., 2016a). In addition, hDPP4 transgenic mice inoculated with MERS-CoV exhibit degenerative neurons and cellular debris which are particularly detected in the brainstem and the thalamus (Tao et al., 2015; Li et al., 2016a). Hence, this suggests that the brainstem and thalamus can invariably be targeted by viral infection regardless of the nature of the CoV (SARS-CoV-1 and MERS-CoV).

Brainstem and thalamic dysfunction that may arise from SARS-CoV-2 infection could be responsible for a large number of diseases diagnosed in exposed patients, especially neurologic symptoms. As an example, the ageusia/dysgeusia that seems to be relatively common in COVID-19 patients (from 5.6% (Mao et al., 2020) to 88% (Lechien et al., 2020)) may very well be the consequence of the dysfunction of these two brain regions. Indeed, the first central relay of gustatory afferents to the brain is the nucleus of the solitary tract, which integrates gustatory information from nerves V, VII, IX and X, and distributes them to the rest of the brain through secondary medullo-thalamic connections (to the posteromedial ventral nucleus of the thalamus) and tertiary thalamo-cortical connections (Heckmann et al., 2003). Therefore, any lesion or disconnection in these brainstem and thalamic regions may result in gustatory dysfunction. It is noteworthy that the brainstem and thalamus are not the only brain regions where viral invasion has been noted in animal studies. In K18-hACE2 mice, SARS-CoV-1 was also detected in the midbrain, hypothalamus, amygdala, hippocampus, basal ganglia, cortex and olfactory bulb (Netland et al., 2008). Therefore, it is possible that these regions may also be dysfunctional following SARS-CoV-1 and SARS-CoV-2 infection and lead to neurologic symptoms.

wBesides pathophysiological aspects, experimental studies raise the possibility that cerebral infection by SARS-CoVs may be one the main trigger of death among patients. Animal studies have demonstrated that intracranial injection of very low SARS-CoV-1 titers leads to rapid death without noticeable lung deterioration (Netland et al., 2008). In addition, intranasal inoculation of low MERS-CoV titers can significantly delay disease onset and reduce mortality rate by 40% in mice (Li et al., 2016a). Under these experimental conditions, death was not associated with any signs of lung infection. Hence, these data suggest that cerebral dissemination of SARS-CoVs may be sufficient to trigger death.

Unfortunately, involvement of any brain lesion or dysfunction in the pathophysiology of SARS-CoV-2, and especially in respiratory failure, is still very speculative given the current state of knowledge. Nonetheless, the existing evidence warrants systematic evaluation of the physical and functional integrity of the brain, especially of the brainstem, in patients diagnosed with SARS-CoV-2, at least those presenting with moderate to severe respiratory failure along with other neurologic symptoms.

## 5 Detection of brainstem dysfunction and neuroinflammation in COVID-19 patients

### 5.1 Predisposing factors and prognosis of neurologic dysfunction

Although the level of evidence indicating a role of microglial priming in post-infectious neuroinflammatory processes is quite low at the moment, extrinsic factors such as pre-existing history of brain injury (traumatic brain injury, stroke, epilepsy, ...) and stress-related diseases may be systematically assessed at admission to complement clinical diagnosis of COVID-19 patients. In addition, long-term neuropsychiatric follow-up of COVID-19 patients exhibiting these potentially predisposing conditions may be recommended.

### 5.2 Detection of brainstem dysfunction in COVID-19 patients

#### 5.2.1 Detection of acute brainstem dysfunction by Auditory Brainstem Responses (ABR) in COVID-19 patients

Because it may spread into the brainstem through peripheral nerves and the olfactory pathway, SARS-CoV-2 could not only affect neural respiratory centers, but also disturb the auditory pathway. Therefore, ABR could be used to assess brainstem injury caused by SARS-CoV-2. ABR belong to the toolbox of ICU and is used to monitor the integrity of brainstem function because they resist well to sedation and muscle paralysis (Stone et al., 2017).

Brainstem dysfunction affects the aspect and the latency of wave V and is associated with prolonged III-V interval, weak (<0.5) amplitude ratio between waves V and I, and depressed wave V (Celesia, 2015). In the most serious cases, wave V could be absent. Hence, the absence of wave V could coincide with a higher probability of brainstem respiratory center injury. Because conductive hearing loss might be observed in patients in intensive care due to middle ear dysfunction, given the absence of swallowing reflexes for long periods of time, tympanometry should be conducted first, to adjust ABR protocols. If conductive hearing loss is objectivized, the level of stimulation in the ABR protocol should be increased, otherwise, wave I would be largely delayed.

#### 5.2.2 Detection of chronic brainstem dysfunction by ABR in recovered patients

Besides acute aspects, potential sequelae of CoV infection may include multiple sclerosis (MS) in patients with genetic predisposition (Burks et al., 1980; Desforges et al., 2019). Post-mortem analyses have demonstrated that the presence of CoV (HCoV-OC43) RNA is relatively common in the brain of MS patients compared to controls (36 vs 14%) (Arbour et al., 2000). Demyelination could be caused by the death of oligodendrocytes, the intense immune response, or the release of inflammatory molecules by infected neural cells. Given the potential neural invasion by SARS-CoV-2, a high incidence of MS among recovered patients might be expected. ABR could be helpful in distinguishing individuals affected by MS after resolution of deadly infectious symptoms. In MS, demyelination induces a heterogeneous slowing of conduction in the auditory pathway resulting in desynchronization. Desynchronization alters ABR by modifying the morphologies of waves III and V, depressing wave V, and lowering to 1 the amplitude ratio between waves V and I. It also disturbs behavioral performance in specific auditory tests (Furst and Levine, 2015). Since integration of binaural information requires high temporal coding performance, psychoacoustic tests like lateralization tests or binaural masking level difference tests are usually disturbed in MS patients. These behavioral tests are low-cost and could be a good alternative to ABR which need specific devices and expertise. Studies with MS cases documented by MRI have shown that behavioral tests are reliable, although they can be time-consuming (Levine et al., 1994; Furst et al., 1995, 2000). Nevertheless, duration for clinical versions of these tests can be shortened (Moore and Sek, 2009; Füllgrabe and Moore, 2017). It is important to note that these behavioral tests have a major drawback, as they can also be influenced by higher cerebral dysfunction (that would, nonetheless, still indicate brain dysfunction in COVID-19 patients). In recovered COVID-19 patients, a longitudinal supervision would improve the efficacy of the screening by comparing the results of ABR and/or behavioral tests to a baseline or to previous follow-up examinations.

### 5.3 Neuroimaging tools for detection of acute and chronic neuroinflammation in COVID-19 patients

Brain inflammatory processes are very difficult to detect and measure. In severely or critically ill patients, lumbar puncture could be performed to measure cytokine levels in CSF. However, this is an invasive and technically challenging procedure. By contrast advanced neuroimaging could be a non-invasive technic to monitor brain inflammatory processes, particularly microglia activation state. Among other techniques, gadolinium-enhanced magnetic resonance imaging (Gd-MRI) or translocator protein-positron emission tomography (TSPO-PET) could be used to detected abnormal activation of brain inflammatory processes in SARS-CoV-2 infected patients (Quarantelli, 2015; Werry et al., 2019). Gadolinium seems to accumulate in inflammatory foci, particularly in the brain of MS patients (Quarantelli, 2015), whereas TSPO ligands can target activated (micro)glia specifically (Werry et al., 2019). Where available, neuroimaging should be prescribed to moderate and severe COVID-19 patients at discharge and as a follow-up examination.

## 6 Conclusion

**Fig. 1:**
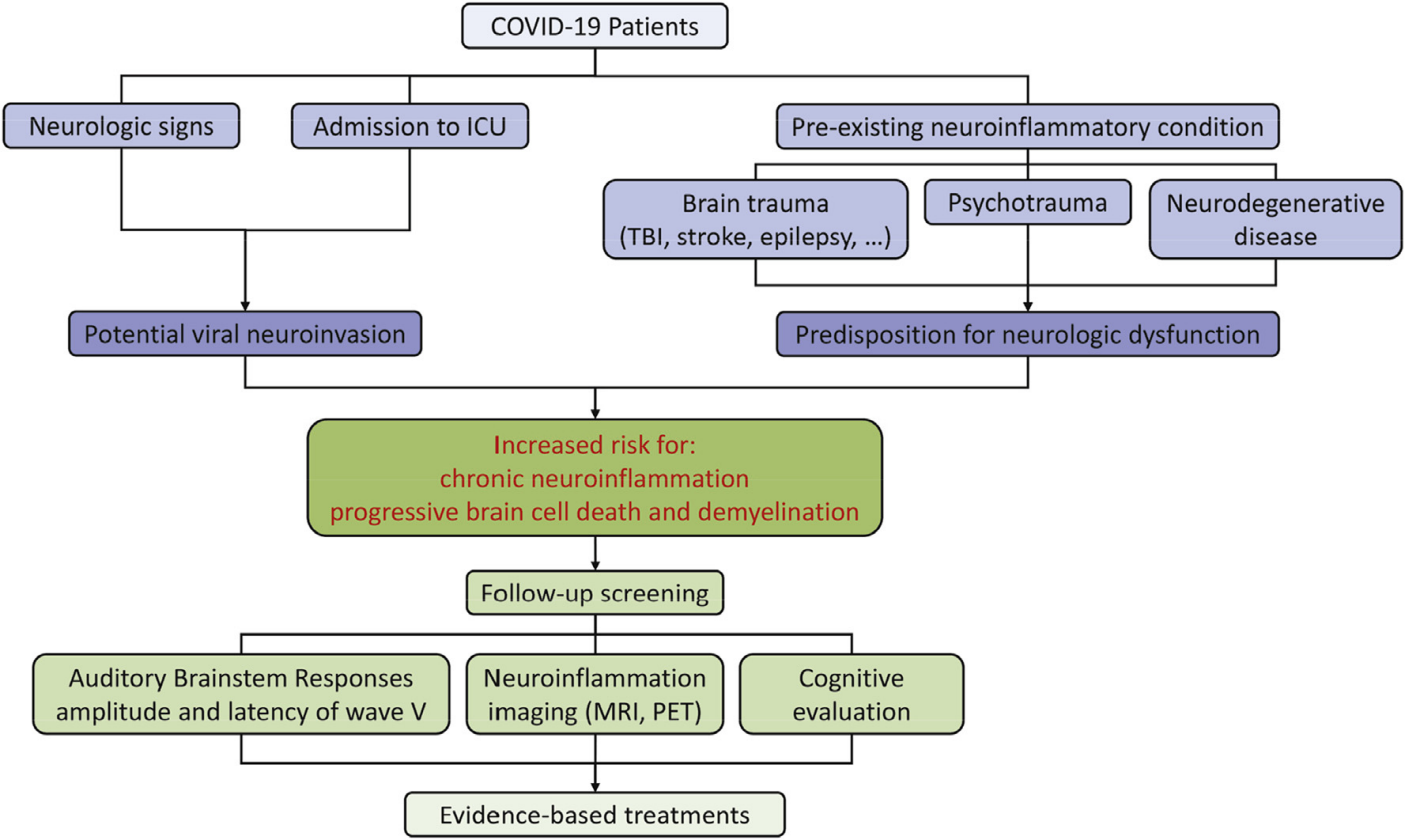
**Long-term follow-up of COVID-19 patients predisposed to chronic neurologic sequelae.** COVID-19 patients that are either admitted to ICU for severe respiratory failure or exhibit neurologic symptoms at initial diagnosis may be considered as having more risks for developing chronic neuroinflammation and brain cell degeneration due to infection. This may also be true for confirmed COVID-19 patients suffering from pre-existing conditions associated with chronic neuroinflammation. Patients exhibiting these characteristics should be submitted to a long-term neurologic follow-up protocol to ensure complete recovery. Auditory brainstem responses, neuroinflammation imaging and routine cognitive evaluation may be used for follow-up examination, as they might help assess existing or emerging brain dysfunction and objectivize further treatment. Abbreviations: ICU, Intensive Care Units; MRI, Magnetic resonance imaging; PET, Positron Emission Tomography; TBI, Traumatic Brain Injury.

Despite the fact that there is still only few evidence, to date, demonstrating that SARS-CoV-2 has potency to invade the brain and induce neurologic defects in patients, previous clinical and preclinical studies conducted on homologous CoV warrant detailed evaluation of brain status in patients infected with SARS-CoV-2. Improvement of the monitoring protocols of patients with ongoing symptoms and those that were, apparently, successfully treated may be implemented in clinical practice (Fig. 1). First, conditions known to be linked with chronic brain inflammation might be screened at patient admission, if possible, or at discharge, to refine diagnosis as well as prognosis of potential long-term neurologic deficits. Second, non-invasive and objective assessment of brainstem function could be performed with ABR in patients in ICU. Third, search for brain sequelae such as MS could be conducted by longitudinal supervision of ABR or specific behavioral tests in recovered patients. Last, when possible, acute and long-term neuroinflammation imaging should be performed to evaluate possible late-onset brain disease.

## Funding source

None.

## Declaration of competing interest

None.
